# Melatonin Has An Ergogenic Effect But Does Not Prevent Inflammation and Damage In Exhaustive Exercise

**DOI:** 10.1038/srep18065

**Published:** 2015-12-16

**Authors:** Wladimir Rafael Beck, José Diego Botezelli, José Rodrigo Pauli, Eduardo Rochete Ropelle, Claudio Alexandre Gobatto

**Affiliations:** 1 Laboratory of Applied Sport Physiology, School of Applied Sciences, Department of Sport Sciences, University of Campinas, Pedro Zaccaria Street, 1.300, Jardim Santa Luíza–Postal Code 13484-350–Limeira–São Paulo; 2 Laboratory of Molecular Biology of Exercise, School of Applied Sciences, Department of Sport Sciences, University of Campinas, Pedro Zaccaria Street, 1.300, Jardim Santa Luíza–Postal Code 13484-350–Limeira–São Paulo

## Abstract

It is well documented that exhaustive physical exercise leads to inflammation and skeletal muscle tissue damage. With this in mind, melatonin has been acutely administered before physical exercise; nevertheless, the use of melatonin as an ergogenic agent to prevent tissue inflammation and damage remains uncertain. We evaluated the effects of melatonin on swimming performance, muscle inflammation and damage and several physiological parameters after exhaustive exercise at anaerobic threshold intensity (iLAn) performed during light or dark circadian periods. The iLAn was individually determined and two days later, the animals performed an exhaustive exercise bout at iLAn 30 minutes after melatonin administration. The exercise promoted muscle inflammation and damage, mainly during the dark period, and the exogenous melatonin promoted a high ergogenic effect. The expressive ergogenic effect of melatonin leads to longer periods of muscle contraction, which superimposes a possible melatonin protective effect on the tissue damage and inflammation.

Melatonin is a pineal gland hormone classically related to entrain the biological rhythms of mammals. Therefore, since Alberti^1^ isolated this hormone from bovines, the spectrum of functions of melatonin has grown. Treatment of health and diseased issues comprises a critical part of scientists´ interest in melatonin^2^,^3^,^4^,^5^, moreover, a considerable number of studies have investigated the effects of melatonin on physical exercise^4^,^6^,^7^,^8^,^9^,^10^,^11^,^12^. According to the literature, even a single dose of exogenous melatonin administered just before the exercise prevents inflammation, oxidative stress and muscle damage^7^,^8^,^11^.

It is well documented that local and systemic inflammation, muscle damage and oxidative stress are induced by long duration or high intensity exercise, impairing skeletal muscle parenchyma function^13^. Some authors have related this effect to deficits in exercise performance^14^,^15^,^16^, encouraging the use of anti-inflammatory compounds to avoid it. Melatonin inhibits inflammation through a variety of stimuli, like *i*) impairing NK-κB-DNA binding^17^, *ii*) inhibiting NF-κB activation^18^ by blocking phosphorylation of IKK and JNK, and consecutive pathways^19^, *iii*) reducing cytokine expression^20^,^21^ and *iv*) acting as an antioxidant and consequently preventing the muscle damage^7^, which also represents important pro-inflammatory feedback.

The effects of melatonin on exercise have been shown in a variety of models^9^,^12^,^22^,^23^; nevertheless, there is no evidence that the protective effects of melatonin persist after long duration exercise performed during both circadian periods. Animal models of swimming performance mimics physiological modulations found in sports competitions with long duration cyclic exercise; however, most laboratory animals are nocturnal. So, the aim of this study was to investigate the effects of melatonin on swimming rat’s performance, skeletal muscle and systemic inflammation parameters, metabolic variables and skeletal muscle tissue damage after exhaustive exercise at an intensity corresponding to the anaerobic threshold (iLAn) performed during the light and dark circadian periods. Based on the literature, we hypothesized that melatonin would contributes as an ergogenic, exerts a protective effect regarding inflammation and tissue damage and improve the physiological status after the proposed exercise during both circadian periods.

## Results

### The LAn intensity was higher in N animals, but lactataemia was not influenced by a time of day effect

The animals assessed in daylight hours showed intensity and lactatemia of iLAn corresponding to 4.8±0.1% of body mass (%bm) and 4.1±0.2 mM, respectively, while nocturnally assessed rats had 5.3±0.1%bm and 4.1±0.2 mM for the same parameters. The iLAn was higher in N (P < 0.01) and lactatemia of iLAn was statistically equal (P = 0.86) between groups.

## Experiment 1

### In the absence of exercise, melatonin did not influence the muscle inflammatory protein content; however, general physiological status was improved

**Figure 1:**
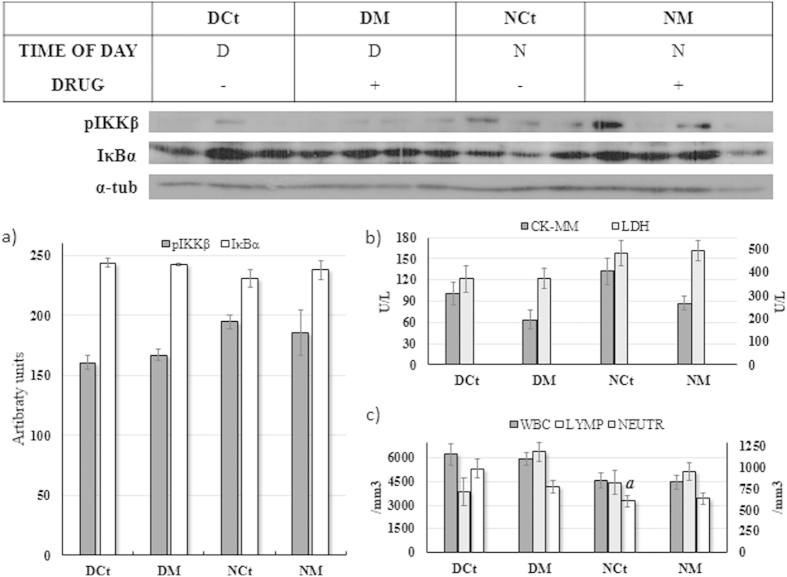
Data from experiment 1 expressed as mean±SEM and significant post hoc results to animals assessed during the daily (DCt and DM) or night (NCt and NM) period and subjected (DM and NM) or not (DCt and NCt) to melatonin administration. Figure 1a shows pIKKβ and IκBα skeletal muscle content. The skeletal (CK-MM, left Y-axis) isoform of creatine kinase and lactate dehydrogenase (LDH, right Y-axis) data are illustrated in Fig. 1b. Figure 1c shows white blood cells (WBC, left Y-axis), lymphocytes (LYMP, left Y-axis) and neutrophils (NEUTR, right Y-axis) results from melatonin and time of day effects. (**a**) P < 0.05 in relation to DCt for same variable.

Figure 1 shows the time of day and melatonin effects on the animals that were not submitted to exercise. Melatonin did not influence either the pIKKβ (F = 0.01; P = 0.91; Fig. 1a) or the IκBα (F = 0.24; P = 0.64; Fig. 1a) skeletal muscle content, and the time of day effect promoted no influence either on the pIKKβ (F = 4.71; P = 0.06; Fig. 1a) or the IκBα (F = 2.25, P = 0.16; Fig. 1a). The exogenous melatonin reduced the skeletal muscle isoform of creatine kinase (CK-MM; F = 7.42; P = 0.01; Fig. 1b) but increased the neutrophil count (F = 5.27; P = 0.03; Fig. 1c). White blood cell (WBC; F = 9.29; P < 0.01; Fig. 1c) and lymphocyte (F = 10.79; P < 0.01; Fig. 1c) count were found to be lower during the period of darkness, while the lactate dehydrogenase (LDH) were found to be higher at this time of day (F = 5.37; P = 0.02; Fig. 1b).

The influence of melatonin and time of day effects on oxidative stress, metabolic and physiological status of serum markers are shown in Table 1. Changes were observed in specific parameters, highlighting that the exogenous melatonin decreased creatinine (CREAT) and UREA, while superoxide dismutase (SOD), catalase (CAT), albumin (ALB), cholesterol (CHOL) and UREA were found to be higher during each rat’s period of wakefulness (darkness).

## Experiment 2

### In the absence of melatonin, exercise promotes acute muscle and systemic inflammation and tissue damage

**Figure 2:**
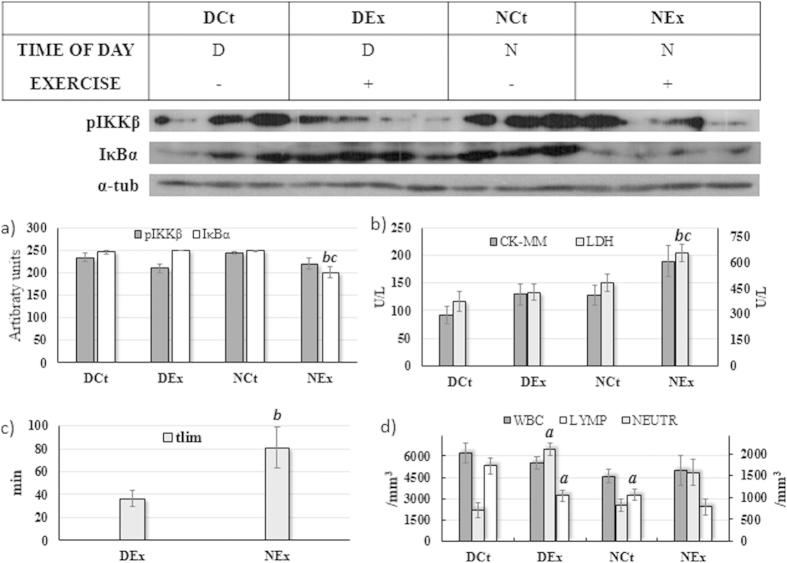
Mean±SEM and post-Hoc results to animals assessed during the daily (DCt and DEx) or night (NCt and NEx) period and subjected (DEx and NEx) or not (DCt and NCt) to the exhaustive exercise at anaerobic threshold intensity. The Fig. 2a shows the pIKKβ and the IκBα skeletal muscle content. The skeletal (CK-MM, left Y-axis) isoform of creatine kinase and lactate dehydrogenase (LDH, right Y-axis) data are illustrated in Fig. 2b. The Fig. 2c illustrate the time to exhaustion at anaerobic threshold intensity accomplished at diurnal or nocturnal periods. The Fig. 2d shows the white blood cells (WBC, left Y-axis), lymphocytes (LYMP, left Y-axis) and neutrophils (NEUTR, right Y-axis) results from exercise and time of day effects. (**a**) P < 0.05 in relation to the DCt group for same variable; (**b**) P < 0.05 in relation to the DEx group for same variable; (**c**) P < 0.05 in relation to the NCt group for same variable.

Experiment 2 analyzed the time of day and exercise effects on animals not exposed to exogenous melatonin. Exercise decreased the pIKKβ muscle content (F = 5.60, P = 0.04; Fig. 2a); however, significant differences were not found between groups. A time of day effect on pIKKβ muscle content was not found (F = 0.96, P = 0.35; Fig. 2a).

The IκBα muscle content was decreased by *t*lim (F = 8.47, p = 0.01; Fig. 2a) and was higher during the day in relation to the night period (F = 10.24, P < 0.01; Fig. 2a), the NEx group having a lower content than all other groups (P < 0.05).

All of the tissue damage markers were found to be significantly increased after the exercise in relation to the control rats (CK-MM: F = 4.77, P = 0.03 and LDH: F = 4.58, P = 0.04; Fig. 2b) and for the animals assessed during the night period (CK-MM: F = 4.47, P = 0.04 and LDH: F = 10.27, P < 0.01; Fig. 2b). The systemic inflammatory parameters (Fig. 2d) showed no time of day (F = 2.40; P = 0.12) or exercise (F = 0.02; P = 0.86) effects on WBC count; nevertheless, the exercise decreased lymphocyte (F = 8.67; P < 0.01) and increased neutrophil counts (F = 25.16; P < 0.01), with only the lymphocytes being influenced by the time of day effect (F = 8.02; P < 0.01; D > N).

Data about exercise or time of day effects on the animals that did not received melatonin are shown in Table 2. Generally, in the presence of exercise, considerable and expected changes were found in several physiological parameters. The exercise decreased the TP, GLOB, CHOL, GLUC and UA, increasing the UREA and CREAT serum concentrations. Higher serum concentrations of CHOL, UREA, AU and CAT were found during the night period; however, the concentration of CREAT was found to decrease during the night.

## Experiment 3

### Melatonin is significantly ergogenic, but did not prevented the exercise-induced inflammation and tissue damage

**Figure 3:**
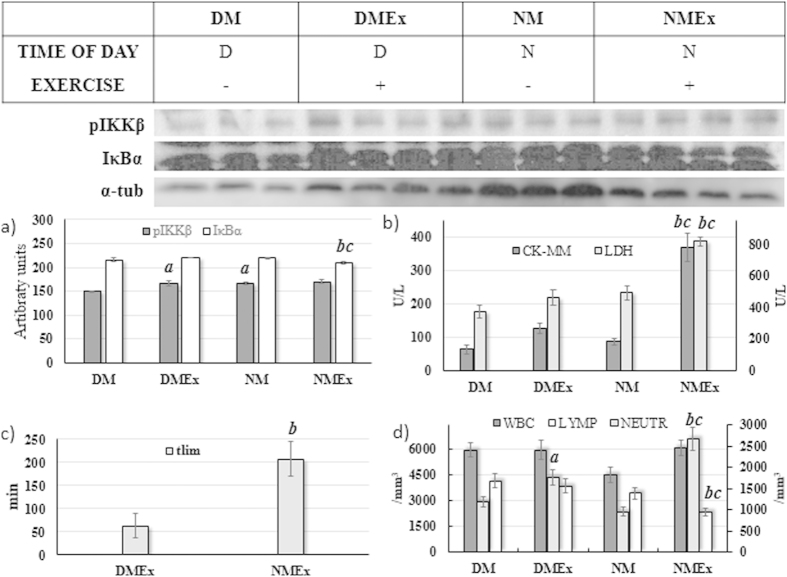
Mean±SEM and post-Hoc results of the animals exposed to melatonin and assessed during the daily (DM and DMEx) or night (NM and NMEx) period and subjected (DMEx and NMEx) or not (DM and NM) to exhaustive exercise at anaerobic threshold intensity. The Fig. 3a shows pIKKβ and IκBα skeletal muscle content. The skeletal (CK-MM, left Y-axis) isoform of creatine kinase and lactate dehydrogenase (LDH, right Y-axis) data are illustrated in Fig. 3b. The Fig. 3c illustrate the time to exhaustion at anaerobic threshold intensity. The Fig. 3d shows white blood cells (WBC, left Y-axis), lymphocytes (LYMP, left Y-axis) and neutrophils (NEUTR, right Y-axis) results from the exercise and time of day effects. (**a**) P < 0.05 in relation to the DM group for same variable; (**b**) P < 0.05 in relation to the DMEx group for same variable; (**c**) P < 0.05 in relation to the NM group for same variable.

This analysis included only animals exposed to exogenous melatonin and subjected to the swimming exercise until exhaustion at anaerobic threshold intensity during both circadian periods. The pIKKβ content was not influenced by the exercise (F = 1.14, P = 0.31; Fig. 3a); nevertheless, higher pIKKβ were found in skeletal muscle during the daily assessments in relation to night (F = 5.18, p = 0.04; Fig. 3a). The IκBα skeletal muscle content was found to be higher during the daily assessments in relation to night (F = 37.06, P < 0.01; Fig. 3a) and the exercise significantly decreased the levels of such inflammatory proteins (F = 19.09, P < 0.01; Fig. 3a).

The exercise led to significant increases in tissue damage markers when compared to the control animals (CK-MM: F = 41.41, P < 0.01 and LDH: F = 24.41, P < 0.01; Fig. 3b), higher outcomes of such parameters being also found at night in relation to the daily period (CK-MM: F = 24.48, P < 0.01 and LDH: F = 31.55, P < 0.01; Fig. 3b). WBC counts (Fig. 3d) were not influenced by time of day (F = 2.22; P = 0.14) or the exercise (F = 2.88; P = 0.09) effects, but lymphocytes (Fig. 3d) were found to be higher in the daylight period (F = 9.44; P < 0.01) and neutrophils (Fig. 3d) were significantly increased by the effect of exercise (F = 40.87; P < 0.01).

Additional data about the physiological and metabolic serum variables in the animals subjected to the exogenous melatonin, time of day and exercise effects are shown in Table 3. Melatonin led to markedly higher performance during the night period (Fig. 3c), leading to differences in the exercised group that received melatonin during the night period (NMEx) in relation to the other groups. The exercise increased the serum concentrations of ALB, UREA, CREAT and UA and decreased TP, CHOL, GLUC and GSH. Higher serum concentrations for the ALB, CHOL, UREA, UA and CAT were found during the night period.

### Comparing all exercised animals, melatonin increased tlim and tissue damage markers

**Figure 4:**
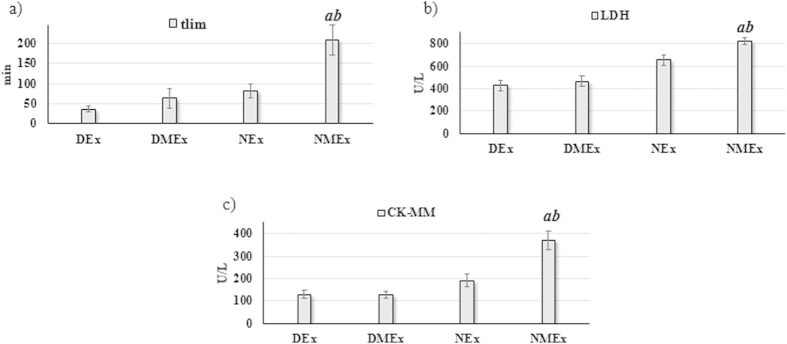
Mean±SEM and post-Hoc results of the time to exhaustion at anaerobic threshold intensity (*t*lim; Fig. 4a), serum concentration of the lactate dehydrogenase (LDH; Fig. 4b) and the skeletal muscle creatine kinase isoform (CK-MM; Fig. 4c) to the exercised groups assessed during the daily (DEx with placebo and DMEx under melatonin effect) or night period (NEx with placebo and NMEx under melatonin effect). (**a**) P < 0.05 in relation to the DMEx group for same variable; (**b**) P < 0.05 in relation to the NEx group for same variable.

Additionally, when the four exercised groups (DEx, DMEx, NEx and NMEx; Fig. 4a) were analyzed, it was found that the melatonin administration significantly increased the time to exhaustion at anaerobic threshold (*t*lim; F = 9.25; P < 0.01). The *t*lim was found to be higher during the night period (F = 14.07; P < 0.01). The NMEx animals showed the highest *t*lim (P < 0.01; Fig. 4a).

The exogenous melatonin increased CK-MM (F = 9.43; P < 0.01; Fig. 4c) and LDH (F = 5.26; P = 0.02; Fig. 4b). Exercised animals assessed during the night period also showed higher CK-MM (F = 27.26; P < 0.01; Fig. 4c) and LDH (F = 42.23; P < 0.01; Fig. 4b) when compared to exercised animals evaluated during the daytime (DEx and DME).

### Resting blood lactate concentration was influenced by time of day and melatonin, but not after exercise

Resting blood lactate levels ([lac]rest) corresponded to 0.93±0.06, 1.29±0.09, 1.47±0.04 and 2.02±0.18 mM in the DEx, DMEx, NEx and NMEx groups, respectively. The blood lactate concentration immediately after exhaustive exercise ([lac]post) was correspondent to 7.01±0.50, 6.75±0.34, 6.93±0.47 and 6.28±0.61 mM to the DEx, DMEx, NEx and NMEx groups, respectively. Melatonin increased [lac]rest (F = 17.32; P < 0.01) and this variable was found to be higher during the night in relation to daytime assessments (F = 33.28; P < 0.01). At rest, the blood lactate concentration of DEx was the lowest (P < 0.05) and NMEx was the highest (P < 0.05). However, the blood lactate concentration after exercise ([lac]post) was not influenced by melatonin (F = 0.80; P = 0.37) or time of day (F = 0.29; P = 0.59) effects, resulting in no differences among groups (P > 0.05).

## Discussion

The main findings of this study were that melatonin has a significant ergogenic effect on the proposed exercise; however, it did not prevented either inflammation or tissue damage resulting from exhaustive exercise. Therefore, the initial hypothesis was only partially accepted, since a preventive effect of melatonin in exercised animals was not found, probably due to its massive ergogenic effect and features of the proposed exercise. As noted during the introduction section, it is well documented that intensity and duration of exercise influence a considerable number of physiological variables. Such a statement was enforced in our experiment, since the *t*lim leads to modulations in almost all of the studied parameters and seems to be confirmed with longer times to exhaustion (Tables 2 and 3).

In order to investigate the effect of exercise on local inflammation, we quantified proteins identified as the master controllers of inflammation^24^, IKK and IκB, responsible for the activity of the transcription factor κB (NFκB). The NF-κB is a key regulator of several biological systems and is associated with linking physiology to pathology when over-activated, its molecular pathway being considered the main inflammatory feedback mechanism in the body^25^. The activation of NF-κB depends on its translocation to the cell nucleus, where it acts in a pleiotropic fashion, influencing a large number of genes^25^. During the resting stage, NF-κB is sequestered in ambient cytosol by κB inhibitors (IκB)^26^, the main inflammatory protein detected in skeletal muscle of adult rats^27^. So, a high IκB content is interpreted as an anti-inflammatory parameter^28^. Stimuli such as high cytosolic calcium concentration^29^ and cytokines^25^,^30^ promote IκB kinase (pIKK) phosphorylation, which is responsible for disconnecting IκBα from NF-κB, eliciting its nuclear translocation and consequently increasing inflammation^30^. In this manner, disconnected IκBα molecules suffer ubiquitination and consequently a decreased level of this protein is found. IKK and IκB phosphorylation is increased by exercise in adult rat skeletal muscle, which invariably leads to local NF-κB activity^27^ and inflammatory feedback activation^31^.

In animals not submitted to exercise (experiment 1), melatonin did not modulate local or systemic inflammatory parameters (pIKKβ, IκBα, WBC and Lymp), but decreased skeletal muscle tissue damage marker (CK-MM, Fig. 2b), creatinine and urea (Table 1), exhibiting the protective features described in the literature^7^,^8^,^11^. However, the main contribution of our study is the interesting results regarding inflammatory and tissue damage markers in animals exercised until exhaustion during the light and dark periods after exposure to melatonin when compared to the control animals.

Despite the ergogenic effect of a single melatonin dose just before acute exercise remaining controversial in the literature^8^,^32^, our study found significantly enhanced performance in the animals that received this hormone (Fig. 2). As described, melatonin presents an anti-inflammatory function and anti-damage musculature effects^7^,^8^,^11^, and performance could be impaired by inflammation and tissue damage^14^,^15^,^16^. However, our results refute such statements in the context of exhaustive aerobic exercise since the systemic inflammation and tissue damage promoted in exercised animals in experiment 3 (all received melatonin) were apparently higher than in animals from experiment 2 (no melatonin). This apparent discrepancy in the inflammatory profile in melatonin treated animals could be associated with the different exercise protocols used in other studies. In addition, we observed that the CK-MM increased by 151.6% in the NEx group when compared to the NCt group, but this outcome in the NMEx group was 324.05% higher than in the NM group. Neutrophil counts, an acute exercise-induced inflammation marker, increased 90.68% in the NEx vs NCt group, but were 180.29% higher in the NMEx group compared to NM. Because the NMEx rats swam 126 minutes (155.84%) longer than the NEx group at the same intensity due to melatonin´s massive ergogenic effect, it is clear why higher inflammation and tissue damage were found in animals that swam longer. Therefore, we introduced this paradoxical melatonin effect on adults swimming rats exercised until exhaustion at anaerobic threshold intensity. Melatonin enhanced the performance but also the inflammation and damage to skeletal muscle tissue. Despite partially denying our initial hypothesis, high levels of inflammation and tissue damage are also found in marathon and ultramarathon athletes, and the relationship between performance and tissue damage and inflammation remains under investigation^33^,^34^.

In general, interpreting all of the physiological, metabolic, oxidative, inflammatory and tissue damage markers assessed in our study, the exercise duration was increased by the effect of melatonin and led to more alterations. Our results suggested that the ergogenic effect of melatonin is significantly stronger than its protective effect with respect to exercise at anaerobic threshold performed until exhaustion, taking the exercise duration as responsible for masking the protective effect of melatonin. Future studies should reproduced this experiment design but employing limits on the exercise duration in order to elicits investigation of the protective effect of melatonin, better understand the mechanistic of melatonin´s ergogenic effect and its role as an anti-inflammatory, antioxidant and tissue damage preventing agent in long duration aerobic exercises.

## Methods

### Animals

Male Wistar rats were housed in polyethylene cages with free access to water and rodent chow, under 12 h light/dark cycle (lights on at 06:00 h), temperature of 22 ± 2 °C, relative humidity kept at 45–55% and noise below 85 decibels. A 100 W lamp was used during the light period (Phillips® soft white light; 2700 K; 565–590 nm; 60 lux). We conducted the experiment according to the current International laws. The study was approved by the Institutional Ethics Committee on the Use of Animals under process 2502-1.

### Experimental design

At 45 days of age, the rats were housed into groups for either diurnal (D) or nocturnal (N) assessments. The specific times of day to begin the procedures were begun were 12:00 and 20:00 h for D and N, respectively, in line with the lowest and highest activity levels of nocturnal entrained rats^35^,^36^. The illumination of the environment was set according elsewhere^37^, employing the under described white light during all of the day period and a red light (15 lux, >600 nm) only during the procedures at night period, in order to avoid light influences on physiological melatonin secretion^38^.

The animals were exposed to two weeks of water environment and swimming adaptation. The procedure was performed in an individual swimming ergometer (cylindrical PVC tank with 30 cm diameter and 100 cm depth, containing clean water at 31±1 °C). Then, at 90 days of age, all the animals were subjected to an incremental swimming exercise test (IT) to determine the intensity corresponding to the anaerobic threshold (iLAn). The IT consists of performing proportional incremental loads over time in order to identify disproportional increases in blood lactate levels at a given moment^39^. So, the animals were submitted to 5-min stages with overloads of 3, 3.5, 4, 4.5, 5, 5.5, 6 and 6.5% of body mass (%bm), as described elsewhere^40^. After each stage, blood samples were collected from the distal part of the tail of each rat to determine the lactate concentration. The exercise intensity relative to the blood lactate concentration was graphically plotted and an alteration in the proportional blood lactate concentration increases was identified by visual inspection, as described elsewhere^41^. Then, two linear regressions were constructed following this break point and the intersection of those linear regressions interpolated to the x-axis was used to define the intensity corresponding to the anaerobic threshold^39^. The interpolation to the y-line corresponded to the blood lactate concentration at iLAn ([lac]iLAn).

Two days after the IT, the rats received an intraperitoneal injection of melatonin and after 30 minutes were subjected to a swimming exercise at iLAn until exhaustion (*t*lim). The melatonin (Sigma Aldrich ©, C_13_H_16_N_2_O_2,_ >98%) was dissolved in ethanol (< 0.1%) and diluted in saline solution (NaCl 0.9%) for administration at 10 mg.Kg^−1^ 42. The control animals for melatonin received the same volume of vehicle (NaCl 0.9%) and the control animals for exercise remained at rest. The control rats were euthanized during the same time of day in relation to the experimental animals. Blood samples were collected before and after *t*lim to determine lactate concentration. The *t*lim was used as the exercise performance parameter.

**Figure 5:**
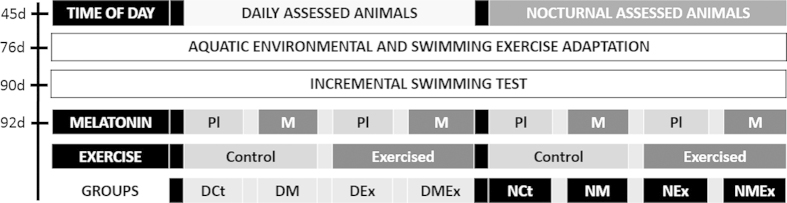
Schematic summary of study design. At 45 days of age (45d) the animals were randomly divided for diurnal or nocturnal assessments. At 76 days of age (76d) begun the aquatic environmental and swimming exercise adaptations. The incremental test was accomplished during the diurnal or nocturnal period according to the groups at 90 days of age (90d). At 92 days of age (92d) *i*) animals were exposed to a vehicle (Pl) or melatonin (M) administration and *ii*) were subjected to the exhaustive aerobic exercise (Exercised) or remained at rest (Control). DCt: daily handling and assessments, vehicle solution, not exercised; DEx: vehicle solution, exercised; DM: melatonin, not exercised; and DMEx: melatonin, exercised. The animals evaluated during the night period followed the same design, using N instead of D for initial acronyms: NCt, NEx, NM and NMEx.

In summary, the animals were randomly divided into eight groups with 15 animals per group: The DCt group (daily handling and assessments, vehicle solution, not exercised); the DEx group (vehicle solution, exercised); the DM group (melatonin, not exercised) and the DMEx group (melatonin, exercised). The animals evaluated during the night period followed the same design, using N instead of D for the initial acronyms (NCt, NEx, NM and NMEx). The experimental design is illustrated in Fig. 5.

Respecting one hour after *t*lim, the animals were exposed to CO_2_ before being euthanized through thoracotomy, and blood extraction was immediately performed by cardiac puncture. The blood samples were divided into two aliquots: *i*) immediately transferred to polyethylene tubes containing k3EDTA (FL Medical, Torreglia, PD, Italy) and *ii*) transferred to empty glass tubes, allowed to rest for 15 minutes and then centrifuged 20 minutes at 3000 rpm to withdraw serum, which was stored at −80 °C for further analysis. The k3EDTA samples were gently mixed by inversion in order to avoid hemolysis and coagulation. The oxidative skeletal muscle soleus was extracted and immediately transferred to liquid nitrogen for subsequent Western Blot analysis. The extraction and storage of all biological material was performed in less than 10 minutes for each animal.

## Analytical Procedures on Biological Material

### Hematological parameters

The hematological parameters were analyzed by hemochromocytometric tests performed on the XS-1000 system for White Blood (Leukocytes; WBC), Lymphocytes (Lymp) and Neutrophils (Neutr) counts.

### Plasma and Serum parameters

In order to determine the blood lactate concentration during the IT, before and after *t*lim, the blood samples (25 μL) were collected from the distal tail of each rat using a micro-heparinized glass capillaries. The blood was immediately transferred to 1.5 mL plastic tubes of containing 400 μL of trichloroacetic acid [4%]. The processed plasma samples were analyzed by an enzymatic method and read spectrophotometrically at 340 nm. The blood lactate concentration was determined by analyzing the samples against a calibration curve that was constructed using five known lactate concentrations, from 1 to 15 mM.

The sera were stored in several aliquots to avoid undesired thaws and were used to determine catalase (CAT), superoxide dismutase (SOD) and total glutathione (GSH) using Cayman Chemical Company-USA assays. Uric acid (UA), glucose (GLUC), total protein (TP), globulin (GLOB), total cholesterol (CHOL), urea, creatinine (CREAT), albumin (ALB) and lactate dehydrogenase (LDH) were assessed using *InVitro* Diagnóstica Ltda-Brazil assays. The skeletal muscle creatine-kinase (CK-MM) isoform was measured using Larorclin Ltda-Brazil assays. All these procedures were performed following the manufacturer´s guidelines.

### Western blotting procedures

The soleus samples were homogenized in ice-cold RIPA Buffer (AMRESCO, OH, USA) with protein inhibitors (100 mmol/L sodium fluoride, 10 mmol/L sodium vanadate, 2 mmol/L phenyl methylsulphonyl fluoride and 0.01 mg aprotinin) employing a polytron PTA 20S generator operated at maximum speed for 30 s and clarified by centrifugation. Protein concentrations were analyzed using the BCA kit (Thermo, NY, USA). A 100 μg aliquot was used to perform Western Blotting analysis as described by Pauli, Ropelle^43^. The pIKK (Ser176; rabbit anti-pIKKβ; 1:1000) and IκBα (C-21; rabbit anti- IκBα; 1:1000) antibodies were obtained from Santa Cruz Biotechnology (Santa Cruz, CA, USA) and α-tubulin (mouse anti α-tubulin; 1:1000) from Novus Biological (NOVUS, CO, USA). Quantitative analysis of the blots was done using Photoshop software (ADOBE, USA).

## Statistical procedures

Data are described as the mean±standard error of the mean (SEM). Since no intervention except time of day was performed until iLAn assessment (D vs N), iLAn and [lac]iLAn were analyzed through the *t*-test for independent samples using pooled data of all animals assessed during the day (DCt, DM, DEx and DMEx) versus night period (NCt, NM, NEx and NMEx). Other data were analyzed for the effects of time of day [day (D) and night (N)], exercise [exercised (Ex) and control (C)] and/or melatonin [melatonin (M) and placebo (Pl)]. In experiment 1, data from western blotting, blood and serum parameters of the DCt, DM, NCt and NM groups were processed through two-way analysis of variance to test melatonin and time of day effects. The DCt, DEx, NCt and NEx groups were used to test exercise and time of day effects in experiment 2 through two-way analysis of variance, while experiment 3 used the DM, DMEx, NM and NMEx groups to test exercise and time of day in animals under the effect of melatonin also using two-way analysis of variance. The *t*lim for experiments 2 and 3 were compared using the *t*-test for independent samples (DEx vs NEx and DMEx vs NMEx, respectively). Additional analyses were conducted for all of the exercised groups (DEx, NEx, DMEx and NMEx) to compare the *t*lim, resting ([lac]rest) and immediately after *t*lim blood lactate concentration through analysis of variance on main effects for melatonin and for time of day. The Newmann-Keuls post-Hoc test was used when appropriate. The criterion for significance was 5%. All statistical procedures were performed using MatLab® 7.0 (MathWorks™).

## Additional Information

**How to cite this article**: Beck, W.R. *et al.* Melatonin Has An Ergogenic Effect But Does Not Prevent Inflammation and Damage In Exhaustive Exercise. *Sci. Rep.* **5**, 18065; doi: 10.1038/srep18065 (2015).

## Figures and Tables

**Table 1:** Descriptive and parametrical statistics results to serum total protein (TP), albumin (ALB), globulin (GLOB), cholesterol (CHOL),glucose (GLUC), urea, creatinine (CREAT), uric acid (UA), superoxide dismutase (SOD), catalase (CAT) and total glutathione (GSH) expressed in mean±SEM for rats submitted to the main effects on melatonin and time of day.

	PLACEBO GROUPS		MELATONIN GROUPS		MELATONIN EFFECT		TIME OF DAY EFFECT	
	DCt	NCt	DM	NM	F	P	F	P
TP (g/dL)	5.81 ± 0.05	5.73 ± 0.06	5.70 ± 0.05	5.53 ± 0.21	<0.01	0.97	0.18	0.67
ALB (g/dL)	2.64 ± 0.11	2.85 ± 0.14	2.17 ± 0.21^a^	3.07 ± 0.11^b^	0.65	0.42	13.54	<0.01
GLOB (g/dL)	3.33 ± 0.08	3.27 ± 0.27	3.37 ± 0.22	2.74 ± 0.19	1.21	0.30	2.37	0.13
CHOL (mg/dL)	81.82 ± 2.30	93.81 ± 3.40^a^	76.86 ± 1.68	101.73 ± 3.05^b^^,^^c^	0.31	0.58	47.23	<0.01
GLUC (mg/dL)	123.68 ± 5.51	124.39 ± 7.85	118.45 ± 7.37	134.53 ± 7.18	0.11	0.74	1.27	0.27
UREA (mg/dL)	59.93 ± 2.59	66.97 ± 1.79	30.36 ± 5.74^a^	63.45 ± 2.18^b^	18.70	<0.01	27.51	<0.01
CREAT (mg/dL)	0.77 ± 0.04	0.59 ± 0.03^a^	0.41 ± 0.05^a^	0.55 ± 0.04^b^	21.34	<0.01	0.23	0.63
UA (g/dL)	5.26 ± 0.23	5.33 ± 0.28	4.43 ± 0.14^a^	5.33 ± 0.30^b^	3.02	0.09	3.98	0.05
SOD (U/mL)	28.88 ± 3.29	32.65 ± 3.46	23.46 ± 3.22	34.10 ± 1.37	0.49	0.49	6.48	0.01
CAT (nmol/min/mL)	15.75 ± 1.25	19.99 ± 1.69^a^	11.99 ± 1.28^a^	22.17 ± 1.01^b^	0.37	0.55	30.89	<0.01
GSH (μM)	25.87 ± 5.31	20.51 ± 2.61	18.96 ± 2.20	25.36 ± 1.89	0.09	0.76	0.02	0.87

^a^P < 0.05 in relation to the DCt group for the same variable.

^b^P < 0.05 in relation to the DM group for the same variable.

^c^P < 0.05 in relation to the NCt group for the same variable.

**Table 2:** Descriptive and parametrical statistics results to serum total protein (TP), albumin (ALB), globulin (GLOB), cholesterol (CHOL),glucose (GLUC), urea, creatinine (CREAT), uric acid (UA), superoxide dismutase (SOD), catalase (CAT) and total glutathione (GSH) expressed in mean±SEM for rats submitted to the main effects on time of day and exercise.

	CONTROL GROUPS		EXERCISED GROUPS		EXERCISE EFFECT		TIME OF DAY EFFECT	
	DCt	NCt	DEx	NEx	F	P	F	P
TP(g/dL)	5.81 ± 0.05	5.73 ± 0.06	5.45 ± 0.04^a^	5.69 ± 0.05^b^	16.03	<0.01	2.27	0.14
ALB(g/dL)	2.64 ± 0.11	2.85 ± 0.14	2.78 ± 0.12	3.02 ± 0.13	1.41	0.24	2.90	0.95
GLOB(g/dL)	3.33 ± 0.08	3.27 ± 0.27	2.69 ± 0.13	2.76 ± 0.08	11.21	<0.01	<0.01	0.96
CHOL(mg/dL)	81.82 ± 2.30	93.81 ± 3.40^a^	77.17 ± 1.60^a^	88.95 ± 1.61^b^	4.42	0.04	27.61	<0.01
GLUC(mg/dL)	123.68 ± 5.51	124.39 ± 7.85	105.77 ± 5.15	107.41 ± 5.77	7.69	<0.01	0.03	0.85
UREA(mg/dL)	59.93 ± 2.59	66.97 ± 1.79^a^	68.41 ± 2.17^a^	78.25 ± 1.99^b^^,^^c^	20.65	<0.01	15.04	<0.01
CREAT(mg/dL)	0.77 ± 0.04	0.59 ± 0.03^a^	0.99 ± 0.10^a^	0.72 ± ± 0.05^b^	8.90	<0.01	13.35	<0.01
UA(g/dL)	5.26 ± 0.23	5.33 ± 0.28	3.73 ± 0.13*a*	4.69 ± 0.22*b*	25.42	<0.01	5.67	0.02
SOD(U/mL)	28.88 ± 3.29	32.65 ± 3.46	38.83 ± 2.72^a^	25.32 ± 1.51^b^	0.23	0.64	3.14	0.08
CAT(nmol/min/mL)	15.75 ± 1.25	19.99 ± 1.69	14.16 ± 1.11^a^	21.55 ± 1.09^b^	<0.01	0.98	20.98	<0.01
GSH(μM)	25.87 ± 5.31	20.51 ± 2.61	18.18 ± 4.59	14.69 ± 0.71	3.11	0.88	1.34	0.26

^a^P < 0.05 in relation to the DCt group for the same variable.

^b^P < 0.05 in relation to the DEx group for the same variable.

^c^P < 0.05 in relation to the NCt group for the same variable.

**Table 3:** Descriptive and parametrical statistics results to serum total protein (TP), albumin (ALB), globulin (GLOB), cholesterol (CHOL),glucose (GLUC), urea, creatinine (CREAT), uric acid (UA), superoxide dismutase (SOD), catalase (CAT) and total glutathione (GSH) expressed in mean±SEM for rats that received melatonin and were subjected to time of day and exercise effects.

	CONTROL GROUPS		EXERCISED GROUPS		EXERCISE EFFECT		TIME OF DAY EFFECT	
	DM	NM	DME	NME	F	P	F	P
TP (g/dL)	5.70 ± 0.05	5.53 ± 0.21	5.71 ± 0.07	5.44 ± 0.06b,^c^	6.34	0.01	0.75	0.39
ALB (g/dL)	2.17 ± 0.21	3.07 ± 0.11^a^	2.61 ± 0.11	3.97 ± 0.30b,^c^	11.78	<0.01	34.23	<0.01
GLOB (g/dL)	3.37 ± 0.22	2.74 ± 0.19	3.06 ± 0.14	2.79 ± 0.51	0.19	0.66	2.40	0.13
CHOL (mg/dL)	76.86 ± 1.68	101.73 ± 3.05^a^	77.39 ± 1.86	91.15 ± 1.44b,^c^	5.74	0.02	84.77	<0.01
GLUC (mg/dL)	118.45 ± 7.37	134.53 ± 7.18	105.05 ± 6.73	98.67 ± 6.37,^c^	11.88	<0.01	0.46	0.50
UREA (mg/dL)	30.36 ± 5.74	63.45 ± 2.18^a^	70.33 ± 1.97^a^	86.29 ± 3.11b,^c^	62.28	<0.01	37.98	<0.01
CREAT (mg/dL)	0.41 ± 0.05	0.55 ± 0.04^a^	0.84 ± 0.04^a^	0.75 ± 0.04,^c^	48.99	<0.01	0.37	0.54
UA (g/dL)	4.43 ± 0.14	5.33 ± 0.30^a^	3.72 ± 0.12^a^	3.83 ± 0.12,^c^	39.78	<0.01	8.21	<0.01
SOD (U/mL)	23.46 ± 3.22	34.10 ± 1.37^a^	33.32 ± 3.16	30.31 ± 2.54	1.32	0.26	2.08	0.15
CAT (nmol/min/mL)	11.99 ± 1.28	22.17 ± 1.01^a^	16.14 ± 1.03^a^	21.39 ± 0.78^b^	2.69	0.11	56.25	<0.01
GSH (μM)	18.96 ± 2.20	25.36 ± 1.89^a^	18.81 ± 2.17	7.35 ± 0.84b,^c^	25.67	<0.01	1.99	0.17

^a^P < 0.05 in relation to the DM group for the same variable.

^b^P < 0.05 in relation to the DMEx group for the same variable.

^c^P < 0.05 in relation to the NM group for the same variable.
